# Effects of postoperative administration of celecoxib on pain management in patients after total knee arthroplasty: study protocol for an open-label randomized controlled trial

**DOI:** 10.1186/s13063-015-1106-2

**Published:** 2016-01-23

**Authors:** Takeo Mammoto, Keiko Fujie, Naotaka Mamizuka, Noriko Taguchi, Atsushi Hirano, Masashi Yamazaki, Satoshi Ueno, Enbo Ma, Koichi Hashimoto

**Affiliations:** Department of Orthopaedic Surgery and Sports Medicine, Tsukuba University Hospital Mito Clinical Education and Training Center, Mito Kyodo General Hospital, University of Tsukuba, 3-2-7 Miya-Machi, Mito, Ibaraki 310-0015 Japan; Department of Anesthesiology, Tsukuba University Hospital Mito Clinical Education and Training Center, Mito Kyodo General Hospital, University of Tsukuba, 3-2-7 Miya-Machi, Mito, Ibaraki 310-0015 Japan; Department of Orthopaedic Surgery, Faculty of Medicine, University of Tsukuba, 1-1-1 Tennodai, Tsukuba, Ibaraki 305-8575 Japan; Tsukuba Clinical Research and Development Organization (T-CReDO), University of Tsukuba, 1-1-1 Tennodai, Tsukuba, Ibaraki 305-8575 Japan

**Keywords:** Multimodal analgesia, postoperative pain, total knee arthroplasty

## Abstract

**Background:**

Multimodal analgesia is achieved by combining different analgesics and different methods of analgesic administration, synergistically providing superior pain relief when compared with conventional analgesia. Multimodal analgesia can also result in reductions in the side effects and complications of analgesia, thereby improving patient safety. Preventive analgesia, treatment before initiation of the surgical procedure, has a potential to be more effective in reducing pain sensitization than treatment initiated after surgery. Multimodal analgesia that includes prophylactic administration of selective cyclooxygenase-2 (COX-2) inhibitors can improve postoperative pain and reduce opioid analgesic consumption after total knee arthroplasty (TKA). However COX-2 inhibitors are not approved for use as preventive analgesia in Japan. Thus, assessing the effectiveness of COX-2 inhibitors during the early postoperative period is important to establish clinical practice guidelines in Japan. This study was designed to examine the effects of celecoxib administration immediately after surgery, in addition to multimodal analgesia, on postoperative pain management after TKA.

**Methods/Design:**

This randomized, prospective, open-label controlled study will include 120 patients undergoing unilateral TKA. All patients will routinely receive single injections of femoral and sciatic nerve blocks, along with postoperative patient-controlled analgesia (PCA) with fentanyl. Patients will be randomly assigned to receive or not receive immediate postoperative administration of celecoxib. The primary outcome is a visual analog scale (VAS) pain score the second day after surgery. Secondary outcomes include opioid consumption, VAS pain score for 7 days after surgery, range of knee motion, evaluation of sleep quality, overall evaluations by patients and physicians, rates of postoperative nausea and vomiting, and consumption of rescue analgesics.

**Discussion:**

The objective of this study is to evaluate the effects of celecoxib administration immediately after surgery on pain after TKA surgery. A randomized controlled trial design will address the hypothesis that administration of oral celecoxib immediately after surgery, along with multimodal analgesia that includes peripheral nerve block and PCA, could reduce VAS pain score after TKA surgery.

**Trial Registration:**

UMIN-CTR 000014624 (23 July 2014)

## Background

Surgical procedure could cause noxious stimulation. Many methods have been used to manage pain after surgery, including various drugs, routes of drug administration and medication methods. Recently, patient-controlled analgesia (PCA) has been more widely used because of its therapeutic effects and safety [1]. PCA is a method of allowing a patient with pain to administer his own pain relief. The infusion is controlled by electronically pump, that delivers prescribed amount of analgesic when a patient press a button. Preventing a patient from overdosing analgesics, a dosage and interval of analgesics are programmed. Patients can self-administer drugs whenever they need analgesics, regardless of broad inter-individual differences in demands for analgesia, thus minimizing inter-individual differences in pharmacokinetics and pharmacodynamics.

Multimodal analgesia is achieved by combining different analgesics and different methods of administration, to provide superior pain relief synergistically compared with conventional analgesia [1]. Moreover, rates of side effects and complications of analgesics are reduced with multimodal analgesia, improving patient safety. The updated 2012 American Society of Anesthesiologists practice guidelines for acute pain management during the perioperative period recommend multimodal techniques for perioperative pain management [1].

Surgical noxious stimuli sensitize the nervous system to subsequent stimuli that could amplify pain. Preventive blocking of nociceptive stimuli to the central nervous system is beneficial in attenuating postoperative pain and in reducing the severity of postoperative pain [2]. Preventive analgesia is a concept for reducing this sensitization before the surgical procedure initiates [2]. Preventive analgesia could be more effective than a similar analgesic treatment initiated after surgery. Surgical trauma induces cyclooxygenase (COX) expression and subsequent synthesis of prostaglandins (PGs), which sensitize peripheral nociceptors and cause nociceptive pain. Since non-steroidal anti-inflammatory drugs (NSAIDs) can inhibit COX and inhibit the synthesis of PGs, NSAIDs are widely used to reduce postoperative hyperalgesia [3].

Traditional NSAIDs inhibit both COX-1 and COX-2 isoenzymes. Celecoxib is a selective COX-2 inhibitor shown to be as effective as traditional NSAIDs as an analgesic for acute postoperative pain. Celecoxib has fewer gastrointestinal side effects than traditional NSAIDs, such as loxoprofen, diclofenac and ibuprofen [4–6]. Moreover, celecoxib has no effects on serum thromboxane and platelet functions, suggesting that it may be an effective postoperative analgesic [7].

Studies have suggested that the administration of selective COX-2 inhibitors for preemptive, multimodal analgesia can improve postoperative pain and reduce the consumption of opioid analgesics after total knee arthroplasty (TKA) [8, 9]. In Japan, however, celecoxib, as well as other NSAIDs, has not been approved for use as preemptive analgesia, but only for postoperative analgesia. Assessing the effectiveness of COX-2 inhibitors as a constituent of multimodal analgesia during the early postoperative period is important to establish clinical practice guidelines.

This study was designed to assess the effects of celecoxib administration during the early postoperative period, along with multimodal analgesia, on postoperative pain management after TKA. We hypothesized that the administration of celecoxib immediately after TKA surgery would provide superior postoperative analgesia compared with delayed administration. Our aim is to compare patient-reported visual analog scale (VAS) pain scores, opioid consumption, range of knee motion, sleep quality, rates of postoperative nausea and vomiting, and rescue analgesic consumption in patients who do and do not receive COX-2 inhibitors, as well as to compare overall evaluations by patients and physicians.

## Methods/Design

### Study design

**Fig. 1:**
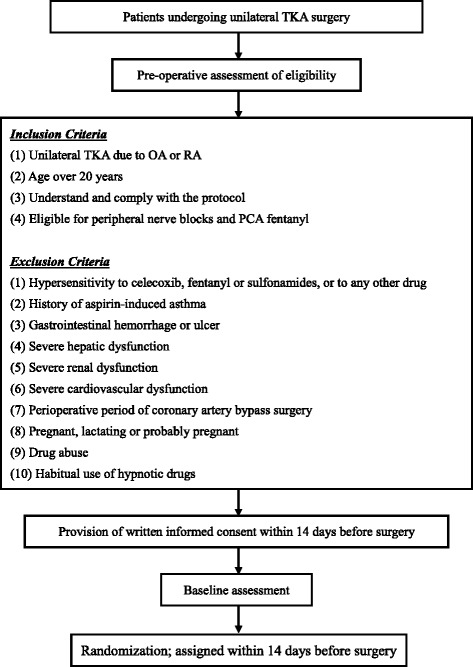
Enrollment: flow diagram of the trial design. Abbreviations: TKA, total knee arthroplasty; OA, osteoarthritis; RA, rheumatoid arthritis; PCA, patient-controlled analgesia

**Fig. 2:**
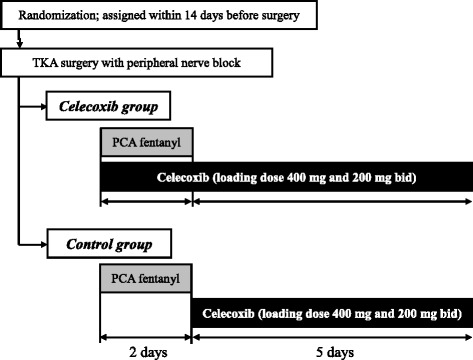
Allocation: flow diagram of the intervention. Abbreviations: TKA, total knee arthroplasty; PCA, patient-controlled analgesia

This study is a single-center, randomized, prospective, open-label controlled trial, performed in accordance with the Declaration of Helsinki and approved by the ethics committee of the Tsukuba University Hospital Mito Clinical Education and Training Center, Mito Kyodo General Hospital, University of Tsukuba. Written informed consent will be obtained from each patient prior to enrollment and testing. This study follows the CONSORT statement (http://www.consort-statement.org/). The trial is registered under the University hospital Medical Information Network and clinical trial registry (UMIN-CTR; http://www.umin.ac.jp/ctr/index.htm) 000014624 (23 July 2014). An overview of the trial design is shown in Figs. 1 and 2.

### Eligibility and recruitment

Patients undergoing unilateral TKA as a result of osteoarthritis or rheumatoid arthritis will be recruited from the Department of Orthopaedic Surgery and Sports Medicine, Tsukuba University Hospital Mito Clinical Education and Training Center, Mito Kyodo General Hospital, University of Tsukuba. Identified patients will be approached for inclusion into the study based on the inclusion/exclusion criteria described below.

### Enrollment criteria

#### Inclusion criteria

Patients will be eligible for inclusion if they are aged ≥ 20 years, undergoing unilateral TKA in the morning as a result of osteoarthritis or rheumatoid arthritis, can understand and comply with the study protocol, have signed the informed consent document at screening, and have been assessed by the principal investigator and/or co-investigator as eligible for pain control with multimodal analgesia (including femoral and sciatic nerve blocks and PCA fentanyl after TKA surgery).

#### Exclusion criteria

Patients with a history of complications or hypersensitivity to celecoxib, fentanyl or sulfonamides; a serious allergic reaction to any other drugs; aspirin-induced asthma; or gastrointestinal hemorrhage or ulcer will be excluded. In addition, patients will be excluded if they have severe hepatic, renal, or cardiovascular dysfunction; are in a perioperative period of coronary artery bypass surgery; are pregnant, lactating or probably pregnant. Patients with a history of drug abuse will be excluded. In this study, sleep quality will be evaluated. Hypnotic drugs could alter the sleep state. The central nervous system active medicines often alter the occurrence, latency of specific sleep and dream state, with either therapeutic intent or side effects [10]. In addition, benzodiazepines are rapid eye movement (REM) sleep-suppressant medications, and could induce a withdrawal syndrome with REM rebound [10]. Therefore, those who take or will take a hypnotic drug from 2 days before surgery to final evaluation will be excluded, although ultrashort-acting hypnotic drugs, including triazolam, zopiclone and zolpidem tartrate, are permitted until surgery.

### Randomization

The objective of this study is to evaluate the effects of additional postoperative celecoxib administration on pain after TKA surgery. All patients will routinely receive a combination of femoral/sciatic nerve blocks and postoperative fentanyl PCA and be randomized using a computer-generated list to one of two groups, one of which will receive immediate postoperative celecoxib (*celecoxib group*), whereas the other will not (*control group*).

A computer-generated scheme will randomize patients in a 1:1 ratio matched by illness, either osteoarthritis or rheumatoid arthritis. Patients will be assigned to either intervention or control group within 14 days before surgery. Randomization will be performed centrally by Tsukuba Critical Path Research and Education Integrated Leading Center (CREIL center), Tsukuba Critical Path Research and Education Integrated Leading Center (CREIL center) Tsukuba Clinical Research and Development Organization (T-CReDO), University of Tsukuba.

### Intervention plan

#### Dosage, route of administration, and period of investigational drugs

In accordance with the package insert, and as in a previous study [9], the first oral dose of celecoxib will be 400 mg, followed by 200 mg in both the celecoxib and control groups. Patients in the celecoxib group will receive 400 mg celecoxib 2 hours after TKA, followed 6 hours later by 200 mg celecoxib and 200 mg celecoxib twice daily beginning the day after surgery until day 7. Patients in the control group will receive 400 mg celecoxib on the morning of the second day after surgery, which is the day of PCA fentanyl removal, followed 6 hours later by 200 mg celecoxib and 200 mg twice daily until the seventh day after surgery [9].

#### Perioperative analgesia

Surgery will be performed under general anesthesia. After general anesthesia is induced, a single injection of peripheral nerve blocks, consisting of 25 ml of 0.375 % ropivacaine hydrochloride as a femoral nerve block and 25 ml of 0.2 % ropivacaine hydrochloride as a sciatic nerve block, will be administered under ultrasound guidance.

Postoperative analgesia will consist of PCA with fentanyl citrate. At the end of surgery, the PCA pump will be started at a basal rate of 10 μg per hour. An on-demand mode will be programmed, allowing for bolus injections of 15 to 20 μg every 5 minutes, with the maximum basal and bolus doses allowed per hour being 250 μg.

#### Rescue analgesic

Patients who require an additional analgesic after PCA fentanyl removal on the second day after surgery will be allowed a 25 or 50 mg diclofenac sodium suppository. Usage of the rescue analgesic will be recorded.

### Outcomes

#### Primary outcome measure

The primary outcome measure in this study will be patient-reported VAS pain score at 9 o’clock on the second day after TKA. This measure will allows us to identify the effect of early administration of celecoxib (Table 1).

**Table 1:** Data collection schedule

	Day before surgery	Day of surgery	Postoperative day(s)				Discontinuance
			1	2	3–6	7	
VAS pain score	●	●^a^	●^b^	●^b^	●^b^	●^b^	●
Fentanyl consumption		●	●	●			
ROM	●			●		●	●
VAS sleep score^c^	●	●	●	●		●	●
SLEEPSCAN™ %	●	●	●	●		●	●
Patient satisfaction						●	●
Evaluation by physician						●	●
PONV		●	●	●	●	●	●
Rescue consumption				●	●	●	●
Adverse events		●	●	●	●	●	●

^a^Two hours after the end of surgery; ^b^9 a.m. before oral administration; ^c^evaluation the next day; % overnight measurement

Abbreviations: VAS visual analog scale; ROM range of motion of the knee; PONV postoperative nausea and vomiting

SLEEPSCAN™ (TANITA Corporation, Japan) is a tool for evaluating sleep quality

#### Secondary outcome measures

For secondary outcome measures, fentanyl consumption through PCA is assessed at 2 days after surgery; VAS pain score is assessed at 9 a.m. each day from 1 to 7 days after surgery; range of motion of the knee joint is assessed at 9 a.m. at 2 and 7 days after surgery; postoperative sleep quality is evaluated at 1, 2 and 7 days; overall patient satisfaction during the period of medication is evaluated at 7 days after surgery; overall evaluation by the physician during the period of medication is assessed at 7 days after surgery; incidence rates of postoperative nausea and vomiting and frequency of taking anti-nausea pills is assessed for the 7 days after surgery; and consumption of rescue analgesics (diclofenac sodium suppositories) after the discontinuation of PCA fentanyl is assessed overall for the 7 days after surgery (Table 1).

Sleep quality at 9 a.m. on postoperative 1, 2 and 7 days will be evaluated by patient-reported VAS sleep disturbance. For objective sleep assessment, sleep latency, sleep efficacy, nocturnal awakening, depth of sleep, body motion, and deep sleep time will be monitored by SLEEPSCAN™ (TANITA Corporation, Japan) [11]. SLEEPSCAN monitors body motion, breathing, and heart rate through the bedding mattress with a water seal and pressure sensor, without the necessity of installing direct sensors, including bioelectrodes, on the human body [11].

### Assessment of safety

Administration of study drugs will be immediately stopped in participants who show relevant deterioration. Severe adverse events, as determined by the Common Terminology Criteria for Adverse Events (CTC-AE) ver. 4.0, will be reported to the principal investigator, who will report any unexpected serious adverse reactions to the institution.

### Data collection

Data collection across time points is shown in Table 1. Data collection is performed by research assistants for VAS pain score, fentanyl consumption, VAS sleep score, SLEEPSCAN, patient satisfaction, PONV, and rescue consumption. Physical therapists will measure knee ROM. Research assistants and physical therapists are independent of the clinical management of the subjects. The collected data will be transferred to the T-CReDO data center as soon as each patient completes the schedules, and will be analyzed by an independent researcher. After collecting data, an independent clinical research coordinator will verify the data collection by confirming medical records. These processes will be unaffected by any researchers.

The validation of collected data for this cohort is important. Previously, patient-reported VAS pain score has been well validated [12–14]. Because of its practicality, reproducibility, sensitivity to treatment effects, and ease of analysis, the VAS pain scale is a powerful research tool in the field of pain research. No significant differences have been observed in VAS pain score for each of the variables including age, sex, and cause of pain [14]. In addition, patients’ satisfaction and sleep quality are also validated previously [11, 15]. Therefore, the collecting data from this study is believed to be validated.

#### Sample size calculation

The primary outcome of this study is patient-reported VAS pain score on the second day after surgery, based on the hypothesis that immediate postoperative administration of oral celecoxib could reduce patient-reported VAS pain score 48 hours after TKA surgery compared with the control group.

Sample sizes are chosen to detect clinically relevant differences using a power analysis based on previously published data evaluating the effect of celecoxib on VAS pain score after TKA [9]. The minimal clinical significance in VAS pain score was defined as the mean difference between current and preceding scores when the subject reported “a little worse” or “a little better” pain [12]. Previous study shows that VAS pain scores 48 hours after TKA surgery were reported to be 21.3 ± 16.8 mm in patients receiving celecoxib plus PCA morphine and 34.3 ± 16.6 mm in patients receiving PCA morphine alone [9]. Based on these findings, we hypothesized that VAS pain score 48 hours after surgery will be 22 mm for the celecoxib group (postoperative oral celecoxib plus nerve block and PCA fentanyl) and 33 mm for the control group (nerve block and PCA fentanyl alone), with a standard deviation set at 20.0 mm and a ratio of patients in the celecoxib to the control group of 1:1. Calculations showed that a sample size of 53 patients per group would provide 80 % power (alpha = 0.05, two-tail) to detect a difference in VAS pain score between the two groups after treatment, and also the minimal detectable change of 10 mm within each group [9]. Assuming a 10 % dropout rate, 60 patients per group, or a total of 120 patients, are required.

### Plan for statistical analysis

The intention-to-treat analysis will be conducted for all patients who underwent randomization. Baseline information on study subjects in two treatment groups such as demographics, preoperative VAS pain scores, ranged of motion and sleep quality will be described. The primary outcome that the differences of VAS pain scores between celecoxib and control groups at the second day after surgery will be analyzed by ANOVA. The secondary outcomes including the pain-medication use, adverse reactions, VAS pain scores, range of motion of knee joint, sleep quality, patient satisfaction, and overall evaluation by physicians after surgery will be analyzed by ANOVA or Chi square test. Nonparametric tests may be used for continuous variables if they are not normally distributed. Between-group comparisons of treatment effect for primary and secondary outcomes, except for pain-medication use and adverse events, will also be conducted with the use of a mixed-effects model, with patient as a random effect and time of assessment (baseline and follow-up days), study group, and baseline values of the outcome as fixed effects.

Significance will be set as *P* < 0.05. All statistical analyses will be performed by using SAS 9.4 (SAS Institute Inc., Cary, NC, USA).

## Discussion

To achieve less postoperative pain, better patient satisfaction, and better rehabilitation after surgery, pain management is very important. Multimodal analgesia for pain management after surgery has been shown effective in controlling postoperative pain [1]. Previously, prophylactic administration of a COX-2 inhibitor, as a multimodal analgesia, has been found to reduce postoperative VAS pain score, opioid analgesic consumption, and active ROM of the knee after TKA surgery [8, 9]. In these studies, a COX-2 inhibitor is administered before surgery.

COX-2 inhibitors and other NSAIDs have only been approved as postoperative, but not as preemptive, analgesia in Japan. In addition, an appropriate fasting period to empty the stomach prior to anesthesia is essential for patient safety, because the risk of inhaling stomach contents into the lungs is reduced. Thus, to verify the effectiveness of administration of a COX-2 inhibitor during the early postoperative period as part of multimodal analgesia, it is necessary to establish clinical practice guidelines. In addition, this method of administration is safe, convenient and general-purpose way in practice. To date, however, no comparative data are available.

This randomized controlled trial design will be used to address the hypothesis, that immediate administration of oral celecoxib after TKA surgery provides superior postoperative analgesia when compared with delayed administration.

The primary outcome of this study is to evaluate the effects of celecoxib administration immediately after surgery on postoperative pain after TKA. Possible benefits of celecoxib include its ease of administration, safety after surgery, its effectiveness and reduction of postoperative opioid consumption. Thus, this study might to identify the influence of immediate administration of oral celecoxib after TKA surgery. This will help to provide clinical management status for patients with TKA surgery.

Opioid consumption for the secondary outcomes will be determined. Opioid is useful and effective for postsurgical pain control. However, the incidence rates of postoperative nausea and vomiting as its side effects could be increased with large amounts of opioid. By taking medicine for pain relief after the early post-operative period, opioid consumption and its side effects could be reduced. As its other expectations, pain relief after surgery might improve the quality of sleep. When postoperative pain is reduced, the range of knee motion with accelerated physical recovery might be improved. Pain relief, good quality of sleep and early functional recovery could improve satisfaction of patients. These will be very important to establish clinical practice guidelines.

The limitation of this study is an open label. However our data will come from patient-reported outcomes and participants will not be affected by any other. Patient–reported outcome will help to understand practical patients’ condition. Therefore, we believe that this study will provide some insight into perioperative multimodal analgesia, which helps to improve perioperative clinical practice.

### Clinical relevance and importance

This randomized controlled trial will help to provide insight to optimize perioperative pain management strategies by evaluating the effect of immediately postoperative administration of analgesics for patient after TKA surgery. These results could improve clinical practice for perioperative pain management and also might help to improve patient satisfaction.

## Trial status

Patient recruitment started in October 2014. The predicted study completion date is March 2016.

## Abbreviations

COX: cyclooxygenase
NSAIDS: nonsteroidal anti-inflammatory drugs
OA: osteoarthritis
PCA: patient-controlled analgesia
RA: rheumatoid arthritis
TKA: total knee arthroplasty
VAS: visual analog scale
